# Wide-Scale Continuous Quality Improvement: A Study of Stakeholders' Use of Quality of Care Reports at Various System Levels, and Factors Mediating Use

**DOI:** 10.3389/fpubh.2018.00378

**Published:** 2019-01-11

**Authors:** Alison F. Laycock, Jodie Bailie, Nikki A. Percival, Veronica Matthews, Frances C. Cunningham, Gillian Harvey, Kerry Copley, Louise Patel, Ross Bailie

**Affiliations:** ^1^Menzies School of Health Research, Charles Darwin University, Darwin, NT, Australia; ^2^University Centre for Rural Health, The University of Sydney, Sydney, NSW, Australia; ^3^The Australian Centre for Public and Population Health Research, University of Technology Sydney, Sydney, NSW, Australia; ^4^Adelaide Nursing School, The University of Adelaide, Adelaide, SA, Australia; ^5^Aboriginal Medical Services Alliance Northern Territory, Darwin, NT, Australia

**Keywords:** quality of care, continuous quality improvement, Indigenous, primary health care, participatory research, dissemination, integrated knowledge translation, evidence use

## Abstract

**Introduction:** Increasing the use of evidence in healthcare policy and practice requires greater understanding of how stakeholders use evidence to inform policy, refine systems and change practice. Drawing on implementation theory, we have used system-focused participatory research to engage diverse stakeholders in using aggregated continuous quality improvement (CQI) data from Australian Indigenous primary health care settings to identify priority evidence-practice gaps, barriers/enablers and strategies for improvement. This article reports stakeholders' use or intended use of evidence at various levels of the system, and factors mediating use.

**Material and Methods:** Interviews were undertaken with a purposeful sample of 30 healthcare stakeholders in different roles, organization types and settings in one Australian jurisdiction and with national participants, as part of the project's developmental evaluation. Qualitative data were analyzed to identify themes and categories relating to use of evidence.

**Results:** Context-specific aggregated CQI data that were relatable to the diverse professional roles and practices provided an effective starting point for sharing perspectives, generating practice-based evidence and mobilizing evidence-use. Interviewees perceived the co-produced findings as applicable at different levels and useful for planning, policy development, supporting best practice and reflection, capacity strengthening and developing new research. Factors mediating use were commitment to best practice; the credibility of the evidence and its perceived relevance to work roles, contexts and decision needs; report format and language; facilitation and communication; competing work pressures and the organizational environment for change.

**Conclusions:** This study found that primary health care stakeholders used evidence on quality of care for a variety of purposes. This could be linked to the interactive research processes used to engage stakeholders in different roles and settings in interpreting data, sharing and generating knowledge. Findings indicate that system-based participatory research using CQI data and iterative, interactive and systematic CQI-based methods can be applied at scale to support concurrent action for healthcare improvement at different system levels. Factors known to influence implementation should be addressed within the research design to optimize evidence use. Further research is needed to explore the utility of interactive dissemination for engaging healthcare stakeholders in informing policy and system change.

## Introduction

There are persistent gaps between research evidence and what happens in healthcare policy and practice (1, 2). Better use of evidence to improve primary health care (PHC) requires understanding of how healthcare stakeholders use research findings to strengthen knowledge, refine care delivery systems and change practice (3), and greater understanding of the factors mediating use.

Knowledge translation literature offers various theories and frameworks relating to evidence use (or non-use) (4). Commonly identified factors relate to the evidence itself and how it is created, intended target audiences, and the context and process of implementation (5). Integrating knowledge translation through different stages of research—a principle common with participatory research (6) and knowledge co-production (7)—is advocated for increasing research relevance and supporting use (8). However, few studies in this field report outcomes, such as use of research (9), and there is little evidence on the outcomes of integrated knowledge translation strategies (10).

### Improving Indigenous Health Through Continuous Quality Improvement and Large-Scale Change

There are wide disparities in health outcomes and life expectancy between Aboriginal and Torres Strait Islander peoples (Australia's Indigenous nations) and the general Australian population (11). Strengthening the use of evidence in PHC is critical to closing this gap in health equity. Continuous quality improvement (CQI) methods (12) have been shown to be effective and acceptable in the Indigenous PHC context (13, 14). They generate practice-based evidence relevant to local care delivery (15) and use participatory approaches that can uphold Indigenous values as expressed in national statements on research and cultural respect (16).

International evidence supports CQI as effective in improving the standard of care, particularly when applied system-wide (17–19). While CQI data are typically used to prioritize and address improvement needs at local and organizational levels (20), they can be aggregated to indicate where improvement efforts are needed at a broader system level. Engaging diverse healthcare stakeholders in the interpretation of aggregated data could be expected to enhance understanding of systems barriers to improving care and health outcomes across populations. However, little research has examined the application of CQI methods—such as systematic data guided activities, designing with local conditions in mind and iterative development and testing (21)—for prioritizing and addressing wider system needs. Such studies can help address knowledge gaps in how to scale-up programs using theory-based approaches (22) and how to use systems approaches that combine dissemination and multi-stakeholder relationships to generate knowledge and address complex health problems (23–25).

These inherent CQI principles, and those of knowledge co-production (26, 27), informed our novel large-scale, systems-focused participatory CQI research project in Indigenous PHC. Our project (Box 1) engaged diverse stakeholders in interpreting regionally and nationally aggregated CQI data from PHC services, with the aim of informing policy and interventions needed at multiple levels of the health system to achieve wide-scale improvement in PHC quality (39). Understanding stakeholders' perspectives and actions in using these CQI research findings is critical to ensuring impact and improving Indigenous health outcomes (40). In this paper, we draw on findings from a developmental evaluation of the project to explore how healthcare stakeholders used (or proposed to use) the research findings, and their perceptions of barriers and enablers to use. We offer insights for researchers and policy-makers about engaging stakeholders with evidence in ways that integrate multiple perspectives, overcome barriers to use and encourage innovative, varied and complementary translation of findings to improve care.

Box 1 **“Engaging Stakeholders in Identifying Priority Evidence-Practice Gaps, Barriers and Strategies for Improvement” (ESP) Project.**

The Engaging Stakeholders in Identifying Priority Evidence-Practice Gaps, Barriers and Strategies for Improvement (ESP) project (28) engaged a range of Indigenous health stakeholders in interpreting regionally- and nationally-aggregated continuous quality improvement (CQI) data. The data were used to identify priority gaps in care, barriers or enablers and strategies for improvement in key areas of clinical care. It aimed to generate knowledge for use in developing policies and strengthening systems and practice to improve the quality of primary health care (PHC) for Indigenous people, leading to improved population health outcomes (29).

**Aggregated CQI Data**

De-identified CQI data were provided by 175 health centers, across five Australian jurisdictions, that participated in the Audit and Best Practice for Chronic Disease (ABCD) National Research Partnership (30). The data were derived from health centres' routine use of evidence-based best-practice CQI audit tools to assess and reflect on system performance, identify improvement priorities and develop strategies appropriate to service populations and delivery contexts (14). The audit tools cover the scope of PHC (e.g., chronic illness care, maternal health, child health, preventive care). The aggregated data used in the ESP project represented over 60,000 clinical audits of patient records and 492 system assessments (31), conducted over a decade.

**ESP Activities/processes**

A phased process used online reports and theory-informed surveys (32–36) to engage stakeholders in disseminating and interpreting the aggregate data from the ABCD National Research Partnership database, and sharing professional and contextual knowledge to develop the ESP project findings. The iterative process was used to analyse, report and disseminate CQI data for chronic illness care, child, maternal, preventive and mental health, and rheumatic heart disease care. The ESP phases included:

Phase 1—Identify priority evidence-practice gaps using most recent analyzed aggregated CQI data

Phase 2—Identify barriers, enablers and strategies for improvement, using reports on trends over time for key indicators relevant to priority evidence-practice gaps

Phase 3—Provide feedback on draft final report leading to Final ESP Report

**Stakeholders at Different System Levels**

The ESP project used existing CQI professional networks and a snowballing recruitment technique to invite the participation of policymakers and managers, members of health boards, clinicians/practitioners and researchers in Indigenous community-controlled and government-operated health centers, peak bodies and support organizations, government departments and research institutions. ESP Project methods are described in detail elsewhere (28, 37, 38).

## Materials and Methods

We draw on the developmental evaluation (41) of the Engaging Stakeholders in Identifying Priority Evidence-Practice Gaps, Barriers and Strategies for Improvement (ESP) project, which used document analysis, surveys, participant interviews and team processes to collect data between 2014 and 2017. The developmental evaluation aimed to inform ongoing project refinement and implementation, explore facilitators and barriers to stakeholder engagement, explore use of project findings and assess the interactive dissemination process used in the ESP project. The developmental evaluation methods are described in detail elsewhere (42).

### Ethics

The study was carried out in accordance with the recommendations of the Australian National Health and Medical Research Council, National Statement on Ethical Conduct in Human Research. The protocol was approved by the Human Research Ethics Committee (HREC) of the Northern Territory Department of Health and Menzies School of Health Research (Project 2015-2329), the Central Australian HREC (Project 15-288) and the Charles Darwin University HREC (Project H15030). All evaluation participants gave written informed consent in accordance with the Declaration of Helsinki.

### Participant Sampling

Purposeful sampling was used to identify and select stakeholders in one Australian jurisdiction in which there was a considerable history of CQI in Indigenous PHC. The sampling strategy allowed for in-depth exploration of diverse and information rich cases (43)—participants included people in policy, management, clinical, CQI and academic roles. Ethics approval enabled inclusion of several participants whose roles encompassed a national perspective on CQI research.

### Data Collection

In-depth, semi-structured interviews were conducted by the lead author (AL) between mid-2015 and early 2017. This timeframe enabled the input of participants involved in different ESP project cycles, and provided evaluative feedback following adjustments to reports and processes over the course of the project. Interviews were conducted face-to-face, by Skype and telephone; all were audio-recorded and transcribed.

The interviews were structured around the aims of the developmental evaluation. An interview guide was developed and piloted and the questions were refined. Example questions relating to the topic areas around use or proposed use of findings, and factors influencing use are provided in Table 1.

**Table 1:** Semi-structured interview questions about engaging with project data and using findings.

**Topic area**	**Example questions**
Discussing and interpreting use	Have you discussed these aggregated data or findings with others? Can you give examples of the outcomes or highlights of your discussions?
Factors influencing use	Are there factors that helped you access and use the reports? Can you tell me how that worked? Have there been barriers to engaging with and using these data? What are they?
Use of data and findings	Can you provide examples of how you have used the aggregated quality improvement data in your practice? Can you describe how the reports of findings have influenced your practice decisions or intentions?
Impact of the project	Would you like to comment on any other impact of the project to date, or impact that you anticipate?

### Data Analysis

Interview transcripts were imported into NVivo10 analytic software (44). Analysis of interview data was undertaken using content analysis (45). Initial readings of the transcripts provided the lead author with a collective overview of stakeholder responses to each interview question. First, all interview data were coded deductively using categories broadly aligned with the research questions and key elements of implementation theory (46, 47): evidence/innovation characteristics, targeted groups, settings, project implementation processes, and use. Then the information coded for each category was re-read and analyzed inductively. A researcher who was not involved in the project independently checked coding in accordance with recommended practice for reliability (48).

Qualitative data on use or proposed use of findings, and factors influencing use, were then obtained from the interview data. The codes arising were compared within and across transcripts, and iteratively refined, until common patterns and themes were identified. The ESP online surveys included questions inviting free text responses about use of ESP data and findings. Responses to relevant questions were read and compared with the coded interview data to check that the codes reflected broader participant perspectives across jurisdictions.

Themes relating to use of findings were not independent of each other; some findings were relevant to more than one theme. We have therefore described the findings according to the predominant theme and the most important influence.

## Findings

Thirty stakeholders were interviewed: 10 clinicians, six academics, five CQI practitioners, five managers and four policy-makers. Some participants held dual roles (e.g., manager/clinician or academic/clinician). Interviewees represented government and Indigenous community-controlled health services (*n* = 17), research/teaching institutions (*n* = 7) and support organizations including regional health networks and peak bodies (*n* = 6). Some participants had engaged in project cycles in several areas of care. Most had worked for some years in Indigenous PHC.

A total of twenty-eight interviews were conducted—26 individual and two small-group interviews involving program teams. Interview length ranged from 23 to 75 min (averaging 50 min). Sixteen interviews were conducted face-to-face—the remaining interviews used Skype or telephone.

Five themes were identified in relation to reported or proposed use of findings from the ESP project: influencing planning and policy; supporting best practice and reflection; capacity strengthening; developing new research, and; multi-level applicability. The factors mediating use were commitment to best practice, perceived relevance, competing work pressures, organizational environment for change, presentation and useability, credibility of research findings, facilitation and communication. The themes are summarized in Table 2 and discussed below. Exemplar quotes and examples illustrating themes and categories are provided in the Supplementary File, with some included in the text.

**Table 2:** Stakeholder feedback on use or proposed use of ESP project findings, and factors mediating use: themes and categories identified.

**Themes**	**Categories**
**USE OF ESP PROJECT FINDINGS**	
Influencing planning and policy	• Targeting high level decision-making • Promoting a strategic approach • Strengthening evidence and opportunities for action • Bringing people together
Supporting best practice and reflection	• Supporting continuous quality improvement activities • Supporting reflection and change • Affirmation
Capacity strengthening	• Building capacity in continuous quality improvement and population health thinking • Developing skills in understanding and interpreting data • Staff orientation
Developing new research	• Developing research based on findings • Using the research methodology
Multi-level applicability	• Influencing change at different system levels • Supporting a systems approach
**FACTORS MEDIATING USE OF ESP PROJECT FINDINGS**	
Commitment to best practice	• Valuing data and evidence • Improving Indigenous health outcomes
Perceived relevance	• To role and work context • Timeliness • Local vs. wider interpretation
Competing work pressures	• Time and workload • Staff shortages and turnover
Organizational environment for change	• Role of managers, organizational change • Primary health care approach v's acute care focus
Presentation and useability	• Report formats for different audiences • Accessible information • Support for learning
Credibility of research findings	• Research history and methodology • Data currency, benefits and limitations
Facilitation and communication	• Interactive process • Methods and paths of communication

*ESP—Engaging stakeholders in identifying priority evidence-practice gaps, barriers and strategies for improvement project*.

### Use of ESP Project Findings

#### Influencing Policy and Planning Change

Interviewees perceived that the ESP project findings were useful for identifying system issues, informing high level policy decisions to drive improvement, directing resources where service performance was consistently poor and advocating for continuing investment in CQI. They were generally regarded as having greater potential for leverage at a higher strategy level than at health center level, including contributing to national policy developments in CQI in Indigenous PHC. Interviewees perceived that the analysis of comprehensive data from Indigenous PHC centers on adherence to guideline-recommended care over time and wide stakeholder input strengthened credibility for these purposes.

Overall, the national findings strengthened arguments for engaging in improvement-focused work, particularly when they aligned with priorities and barriers observed in organizational and local contexts (e.g., health center-community links, staff training in chronic illness management). Some interviewees regarded CQI data use as useful for fostering closer engagement between people in different roles, for example between policy-makers and practitioners.

“*I see the role of data as actually bringing strategic people much closer to the frontline practitioners, and then saying to frontline practitioners, ‘We need to do something about this, help us and we will help you’.”* (Manager 2)

Interviewees commonly expressed concern about the increasing prevalence and earlier onset of preventable chronic conditions among Indigenous people. A number suggested the findings could inform a more comprehensive PHC model, with greater community involvement, a less siloed approach to care delivery and more efficient use of resources. Use of the findings in combination with other available evidence was commonly reported or proposed. For example, using identified barriers to addressing priority evidence-practice gaps: to help explain jurisdiction-level key performance indicator results; to build on information collected through CQI and dialogue between managers, PHC teams and communities for regional planning; and to enrich team discussions and planning to address improvement needs identified through CQI processes in health centers.

#### Supporting Best Practice and Reflection

The ESP process required stakeholders to use the findings from the ESP study on priority evidence-practice gaps to reflect on and identify barriers, enablers and strategies for improving care (through ESP survey responses). Interviewees in CQI roles reported using the ESP reports in their work with health teams.

“*I'm using them. I used them in some feedback recently—you know, ‘This is a summary of research … we already know these clinical datasets or morbidity. This is the evidence. These are the things that have been measured, this is what we have been looking at and this is what they have foundș… for me, it's very useful.”* (CQI practitioner 2)

Dissemination of the ESP study reports led to both opportunistic and scheduled conversations about improving care, particularly in workplaces where CQI was embedded. Findings often concurred with interviewees' local experiences, reinforcing that other teams were experiencing similar challenges and offering ideas for addressing them. Some participants used the findings as affirmation that their commitment and efforts were worthwhile and could make a difference in improving health outcomes.

For some, the ESP findings prompted immediate action (e.g., checking that items of maternal care were included in the health service template). For many, they sparked reflection on broader issues, such as the impact of social determinants on health, the interdependencies of priorities and barriers identified through the research and the importance of sharing health center data with policy makers. Reflection on the important role of Indigenous staff for improving care quality and cultural safety, and the need to strengthen preventive care and improve the quality of life for people with chronic illnesses, were commonly reported.

#### Capacity Strengthening

Interviewees said that the ESP reports provided resources for CQI training in understanding data and population health, undergraduate clinician education and orientation of new health researchers to CQI. Some used the results for comparison with their local data to stimulate discussion of improvement barriers and strategies. In one instance, ESP results were used to justify a proposal for a community engagement and education program.

Outcomes reported by interviewees included better understanding of: using data to inform decision making; how CQI can work at multiple healthcare system levels; improvement processes and strategies, and; the value and interpretation of box-and-whisker-plot graphs (which display the distribution of data based on the five-number summary: minimum, first quartile, median, third quartile, and maximum). The value of sharing ideas and collaboratively producing knowledge through the project processes was widely acknowledged.

“*They've provided another layer of information that's stimulated thinking and discussion, that's brought in knowledge and expertise and experience from a broad group. It's really enriched the work that we've done.”* (CQI practitioner 1)

#### Developing New Research

Researcher interviewees described ways in which the findings influenced or informed their work, reinforced existing evidence or helped to document failings. Several reported using the findings in successful grant applications for quality improvement research. In one example, the ESP research design was used as a model for community-based research, with the graphs informing the research team's analysis and reporting of data. In another example, the ESP methodology was applied when supporting a health service to undertake a CQI project. It was proposed the findings be used by researchers and clinicians to co-design intervention research addressing specific priorities.

#### Multi-Level Applicability

As described above, the reports were perceived by interviewees as useful for focusing on broader PHC and CQI issues, and for sharing information and knowledge between people at different levels of the health system. The potential for the ESP findings to influence change at multiple levels (national, jurisdiction or regional systems, health center and community level) was widely acknowledged, with the reports seen as providing evidence to (1) shift government policy and strategy, (2) influence organizational planning, (3) stimulate team discussions and decisions, and (4) support practitioners' practices to improve care. The need for complementary strategies at different levels was identified, e.g., policy and staff training to improve access to culturally appropriate services, effective staff recruitment and retention strategies and better coordination of care services. Several interviewees referred to the findings as demonstrating the value of a systems approach for improving care.

“*You can just see how fixing systems for one area of care, such as childhood anemia, would work across other areas of care.”* (Academic 2)

### Factors Mediating Use of ESP Project Findings

#### Commitment to Best Practice

Commitment to providing best practice care and improving Indigenous health outcomes was reported to be a strong motivator for the use of ESP findings. Interviewees generally spoke of the desire to use relevant evidence to improve individual and team practice, deliver better PHC services, and make a difference. Interviewees in clinical and CQI roles talked about using the findings to “work smarter” and strengthen their understanding of how to improve the health of people accessing their services. Comments from policy and senior management perspectives reflected the same motivation for higher-level strategic use. Interviewees in various roles spoke of the value of the data for aligning intentions and practices.

“*I want best practice. I like to be able to measure that. I'm comfortable with data, and I'm wanting the very best practice—looking at data and wanting to understand where we've been, where we are and where we want to go*.” (Clinician 3)

#### Perceived Relevance

Interviewees in a range of Indigenous PHC roles and contexts found ESP findings on improvement priorities and barriers relevant to their work—they validated other evidence, affirmed the real-life experiences of Indigenous clients and PHC teams and provided useable knowledge. Further, the aggregated data used in the research represented the work of PHC staff in recording and auditing client care. Timing was an important factor: when report publication coincided with immediate information needs it supported opportunistic use.

Those with an understanding of population health and experience in CQI appeared to engage more readily with the data. Some perceived the research reports as most useful for staff in specialized programs (e.g., chronic conditions programs) than for generalist staff, because they focused on specific areas of care. Others were uncertain about who the research targeted and who would take responsibility to use the findings. Several interviewees referred to the need for regional and local contexts to be considered when using the system-wide ESP project findings to inform policy or plan interventions.

“*You do need to be a bit careful that a national report hides important jurisdictional differences, and that national decisions are made without reference to more detailed data, which would inform a more locally responsive answer to a system issue.”* (Policy practitioner 2)

Several interviewees suggested that researchers, health center staff and policy makers may have different perceptions of what comprises potentially powerful evidence to take to high-level policy and funding groups.

#### Presentation and Useability

Presenting research findings in 1:3:25 report format (1-page key messages: 3-page summary: 25-page detailed report), accompanied by a plain language summary and data supplement was widely thought by interviewees to offer “something for everyone.” The key messages for action (in the final reports) were considered practical for focused team conversations about improving care. Generally, those with more research or data experience sought the detailed information provided in the full reports. Plain language summaries were thought to be vital for use by a range of staff—more so after the inclusion of explicit statements about how the findings could benefit people in different roles. Several interviewees suggested summaries provided a pathway into the full reports.

“*I think it's getting the information out there in easily digestible form, so that people have an option to look at a summary that says these are the key areas or can drill down and look in more depth—because people in different roles want different levels of information*.” (CQI practitioner 1)

Interviewees perceived that box-and-whisker-plot graphs were valuable for effectively presenting data on trends and variation in care delivery across health centers. Some were concerned that the level of understanding required to interpret these graphs discouraged engagement—the accompanying interpretation guide was important. ESP reports were compared favorably with other web-generated reports of analyzed audit data that services received, because they reported collaboratively interpreted (rather than just analyzed) CQI data.

#### Competing Work Pressures

Managing competing work demands and being time poor were dominant issues. Many interviewees described the nature of work in Indigenous PHC settings as a barrier to engaging with the findings. High staff turnover and staff shortages were commonly identified as contributing to work pressures in remote PHC settings and organizational management. The challenge of being able to sustain planned improvement efforts based on the findings was raised in relation to staff turnover.

#### Organizational Environment for Change

Managers were perceived to have an important role in engaging with the findings and setting the agenda so that “everybody comes on board.” The challenge of facilitating and sustaining change within health centers and teams was noted. Several participants talked about the difficulty of getting traction for implementing quality improvement at a time when managers were engaged in organization restructuring. Managing high acute care workloads in PHC settings was also identified as a factor impacting negatively on uptake of findings.

#### Credibility of Research Findings

Most interviewees with considerable experience working in Indigenous PHC were aware of the achievements of the ABCD CQI research program and many were aware of the establishment of the multidisciplinary CQI research network through which the ABCD data and ESP project findings were disseminated. The program's aim and longevity, evidence base, collaboration between community-controlled and government operated services and applied nature were regarded positively. This influenced regard for the ESP project, also noted for its participatory approach and use of up-to-date CQI data. These features were linked to the significance of the findings for improving PHC quality for Indigenous communities, including their potential to enrich local CQI activities and routine practice.

#### Facilitation and Communication

Participants spoke positively about the way the interactive ESP dissemination process engaged stakeholders in data interpretation and knowledge exchange, acknowledged input and provided opportunities for checking and further input prior to finalizing results.

“*I do like [the reports] presenting the stakeholder priorities back to the stakeholders when asking about barriers and enablers. “This is what you've said, we've taken that on board. This is the next step. What can we do about it?” I think that's really powerful to acknowledge the consultation and to reassure people that their voices have been heard.”* (Academic 3)

The phased ESP research design was likened to a participatory Plan-Do-Study-Act CQI cycle for the way it linked the data, improvement priorities and strategies. Repeated opportunities to engage in project phases was reported as facilitating deeper understanding of the ESP data, and how data could inform decision making. When recounting or proposing use of findings, participants often described interactive processes (e.g., facilitated discussions, education sessions, collaborative planning, stakeholder-researcher dialogue). People in CQI roles were important facilitators of these processes and in disseminating the ESP reports. Interviewees suggested that forums increasing engagement with ESP findings include quality improvement collaboratives.

Acknowledging that people access, understand and assimilate information in different ways, some participants suggested communicating the findings in non-written formats, such as webinars and video-clips. Other suggested mechanisms were newsletters, institution websites, professional association and network websites and online information repositories.

## Discussion

### Summary of Findings

**Figure 1:**
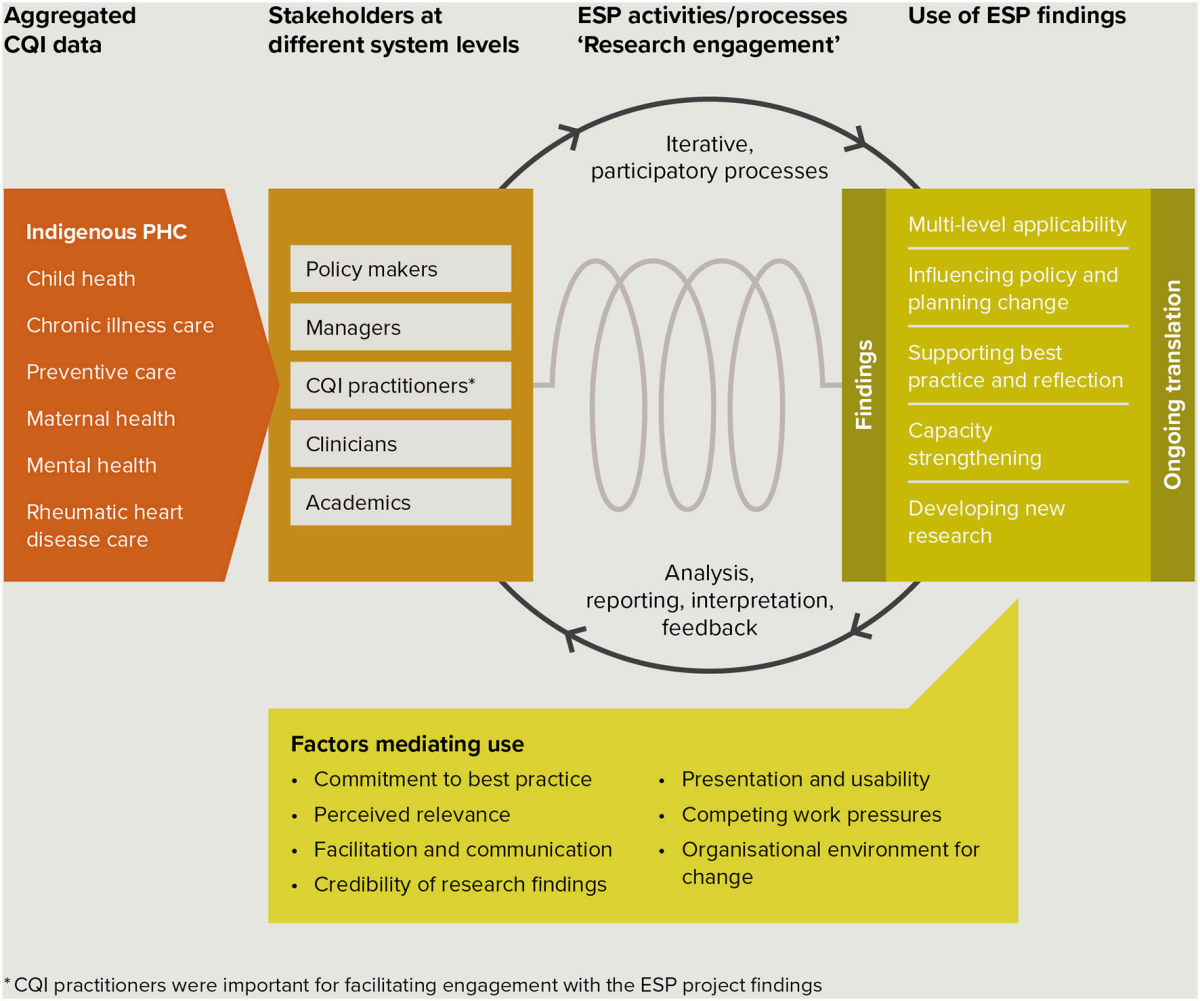
Schema of key study findings in the context of the ESP project. ARF/RHD, Acute rheumatic fever/rheumatic heart disease; CQI, continuous quality improvement; ESP, “Engaging Stakeholders in Identifying Priority Evidence-Practice Gaps, Barriers and Strategies for Improvement”;^*^CQI practitioners were important for facilitating engagement with the ESP project findings.

Interviewees used the CQI research findings to inform policy, practice, capacity development and research. Use was mediated by factors relating to individual motivations, the way evidence was perceived and presented, the context for use and interactions with others. The schema at Figure 1 summarizes key study findings in the context of the ESP project. Interviewees representing stakeholders at different levels of the health system described (1) use of ESP findings derived from interpreting aggregated CQI data in different areas of care, and (2) factors mediating use in the Indigenous PHC context. The schema illustrates the way in which the translation of findings was supported through ESP activities and iterative processes through which analysis, reporting, interpretation and feedback were integrated into the research, with the aim of encouraging ongoing use in policy and practice.

### Interpretation and Comparison With Existing Literature

Context-specific aggregated data derived from the use of evidence-based best practice CQI tools provided an effective starting point for mobilizing evidence-use across different stakeholder groups. It provided interviewees with opportunities to reflect on and discuss practice-based evidence relevant to their experience in the Indigenous healthcare sector, and to consider findings on system-wide improvement priorities and barriers in relation to regional and local evidence. Practice-based research findings are likely to be more relevant, believable, and actionable to practitioners (15). The variety of reported and intended uses for the ESP findings are consistent with the diversity of roles and perspectives represented in the sample, and the participatory principles of CQI in Indigenous PHC (14, 30) that informed the research design.

The integration of knowledge translation into iterative research processes resulted in different types of evidence use (49). At least three types of evidence use are commonly described in the literature—conceptual use that increases knowledge and understanding, instrumental use with a practical focus for bridging evidence-practice gaps, and strategic use (50, 51). All were reflected in our results. ESP findings were used conceptually and instrumentally to support work-relevant learning (52) and improvement (e.g., by CQI practitioners and educators). Strategic use was described by policy-makers. A further type, “process use” (53, 54), was demonstrated when changes in thinking and procedures resulted from learning that occurred during involvement in the research (e.g., improved understanding of CQI data prompted a PHC team to improve recording of client care). Our research methods also enabled the use of different forms of information—aggregate data, research findings and the ESP research methodology—and continuing collaboration of diverse stakeholders through implementation research based on ESP findings.

The study findings are also consistent with studies suggesting that evidence use reflects the system levels at which participants work and the information needs and functions of professional roles (51, 55). People working at each level used the findings in conjunction with other evidence, indicating that they actively sought and used evidence to improve their practice.

The factors mediating use within our sample generally reflect factors identified in Greenhalgh and colleagues landmark review of the diffusion of service innovations (3) and in implementation frameworks (4, 56) and studies. Common factors included the perceived merits of the evidence (57, 58), its relevance to tasks in context (59) and its potential for needs-specific modification (e.g., use of ESP reports in different ways). System readiness (e.g., environment for change, available time) and compatibility with individual values and motivation (e.g., commitment to best practice) were influential. Other mediating factors were the level of implementation support (e.g., from managers and CQI facilitators), continuing communication and tailored information products (55, 60, 61) and opportunities for interaction, supporting the common understanding that research use is social, interactive and context-dependent (62, 63). While lack of confidence in analyzing data was perceived as a barrier to research participation, interviewees in our study were less inclined to identify knowledge and skills as mediating the use of ESP findings. Linkage between the ESP project and stakeholders (a central component in Greenhalgh and colleagues' conceptual model) (3) occurred through the ABCD National Research Partnership in the design stage and was implemented through the research team, with CQI opinion leaders and practitioners playing an important facilitation and boundary spanning role (3). CQI practitioners worked across boundaries to disseminate reports, facilitate participation in the research, promote and discuss findings in PHC services and encourage comparison of aggregated and local CQI data. These activities supported researcher-stakeholder linkage and finding-informed decision-making (64), contributing to the variety of reported and proposed uses of findings. Our study thereby established that participatory research informed by implementation and CQI theory can be applied effectively at scale to engage diverse stakeholders in using evidence on quality of care. It can work across boundaries and system levels to create synergy for improvement.

### Implications for Knowledge Translation Practice and Future Research

These findings can contribute to debate about enhancing the useability and impact of research (26, 65), including the intensity of engagement required between stakeholders and researchers. First, the processes used to successfully engage stakeholders with data and findings were informed by implementation research suggesting that effective change strategies can be developed using evidence to identify and link priority gaps in care to theoretical domains that are known to be system enablers or barriers (32, 33, 66). Second, the varied uses of findings by diverse stakeholders at different system levels were achieved through an interactive dissemination strategy that used high-level aggregated CQI data. It thereby challenged conventional approaches limiting the feedback and use of health center performance data to local CQI activities, demonstrating its potential for planning policy and system-level improvement efforts. Third, the strategy incorporated use of findings at each phase (e.g., participants used findings on barriers to suggest strategies), and this design appeared to positively influence use of the final reports. Fourth, online dissemination and feedback were effective for engaging stakeholders in evidence co-production and use at scale. This demonstrates that engagement can be achieved with limited resources for research when the research and findings are sufficiently practice-relevant and there is time to foster participation. Finally, a multi-disciplinary CQI research network (the Center of Research Excellence in Integrated Quality Improvement in Indigenous Primary Health Care) (67) provided a suitable platform for interactive dissemination and engagement with the findings.

Interviewees represented a sample of ESP project participants. Their project “buy-in,” perspectives on use of the findings to improve care and their generally high regard for the data source and research quality augur well for continuing and wider use of findings (68). However, considerable feedback related to proposed rather than reported use. Descriptions of the uptake of research findings into healthcare as haphazard, unpredictable and messy (5, 69) acknowledge that many factors are likely to interfere with realization of intentions (70, 71). Studies to establish positive correlation between the intentions and behaviors of health professionals pose methodological challenges but are encouraging (72). Acknowledging that longer term impact will require a continuing knowledge translation strategy (73–75), we posit that because the ESP project findings were co-created by researchers and other stakeholders, encompass explicit and tacit dimensions of knowledge (58) and reflect organizational, community and cultural contexts (65), they match the needs of health services, policy makers and affected populations and are more likely to be “owned” and used for change (76).

The ESP project offers promising methods for involving stakeholders in generating knowledge to inform policy and multifaceted improvement strategies (77). The broad-scale and multi-level focus of our CQI research responds to previous work identifying the need for concurrent change at multiple levels of the health system to support wide-scale improvement (39) and the involvement of multiple types of stakeholders to implement and sustain CQI efforts (78). It demonstrated that a structured data and knowledge sharing process and shared commitment to improving Indigenous health outcomes could productively connect stakeholders from different professional perspectives and PHC contexts. Capturing these perspectives to identify priority evidence-practice gaps, and enablers and barriers to addressing them, enables future policy and research to focus on areas important to people involved in Indigenous PHC delivery. Interviewees recommended that ESP findings be used to influence resource allocation and develop translation strategies targeting high-level policymakers. This reinforces our recommendation that further research should explore the utility of interactive dissemination for engaging stakeholders in informing policy and system change.

### Strengths and Limitations

This qualitative study captured the perspectives of people working at different health system levels, in various roles and organization types and using CQI research findings relevant to different areas of clinical care. It explored the implementation of evidence to improve care, helping to address concern that CQI activities in the Indigenous PHC sector have tended to focus on data collection and auditing, with less emphasis on data interpretation and implementing interventions (79).

The interviews focused primarily on one Australian jurisdiction in which CQI is well-established. Stakeholders in jurisdictions with less CQI experience may have different perspectives and experiences of CQI, limiting generalisability. This limitation is offset by inclusion of several interviewees in cross-jurisdiction roles (e.g., a national support organisation), and by comparing interview data with qualitative ESP survey data from other jurisdictions to check the generalisability of findings.

## Conclusion

This study in the Australian Indigenous PHC context identified the use of research findings by stakeholders in a variety of roles and at different health system levels, and factors mediating use, over the course of a large-scale participatory CQI research project.

Context-specific aggregated CQI data provided an effective starting point for sharing perspectives, generating practice-based evidence and mobilizing evidence-use. While factors mediating use were generally consistent with previous studies, engaging stakeholders in participatory processes to interpret the data resulted in different types of use that could be considered complementary, and strategies that were tailored to work needs and applicable at different system levels. CQI methods provided an iterative, systematic and interactive dissemination process that was feasible at scale.

The system-based research design and translation process have implications for using CQI data and approaches in policymaking to create synergy for, and advance, wide-scale healthcare improvement. Increased effort and research are needed to support the use of aggregated CQI data in this way.

Large scale improvement efforts involve long timeframes and planned approaches. Ongoing translation of the ESP project findings into policy and practice to improve Indigenous health outcomes will require the collective commitment and sustained involvement of multiple healthcare stakeholders at different levels of the PHC system.

## Data Availability Statement

The qualitative data analyzed for this manuscript are not publicly available because of the need to protect the identity and confidentiality of research participants.

## Author Contributions

AL conceptualized the study and led the data analysis, interpretation, writing of drafts, and finalizing the manuscript. RB leads the ESP project and supervised the writing process. JB, NP, FC, and GH provided advice during manuscript development. JB and VM contributed to the ESP project design. KC and LP assisted in ESP report dissemination and recruitment of study participants. All authors reviewed drafts, read, and approved the final manuscript.

### Conflict of Interest Statement

The authors declare that the research was conducted in the absence of any commercial or financial relationships that could be construed as a potential conflict of interest.

## Abbreviations

ABCD: audit and best practice for chronic disease
CQI: continuous quality improvement
ESP, engaging stakeholders in identifying priority evidence-practice gaps: barriers and strategies for improvement
HREC: Human Research Ethics Committee
PHC: primary health care.
