# Supplementary material for: Wide-Scale Continuous Quality Improvement: A Study of Stakeholders' Use of Quality of Care Reports at Various System Levels, and Factors Mediating Use
Source: Front Public Health. 2019 Jan 11;6:378. doi: 10.3389/fpubh.2018.00378 (PMC6338065; doi:10.3389/fpubh.2018.00378)
Supplement: Supplementary file 1 [file Data_Sheet_1.pdf]

# *Supplementary Material*

## Wide-scale continuous quality improvement: A study of stakeholders' use of quality of care reports at various system levels, and factors mediating use

*Laycock, A[1], Bailie J[2], Percival N[3], Matthews V[2], Cunningham F[1], Harvey G[4], Copley K,[5] Patel L[5], Bailie R[2]

*Corresponding author: Alison Laycock, alison.laycock@menzies.edu.au

### Exemplar quotes and examples relating to stakeholders use of ESP project findings and factors mediating use

#### Use or proposed use of ESP Project findings: themes and categories with exemplar quotes, examples of use

| Category | Exemplar quotes | Examples |
|---|---|---|
| **Theme: Influencing policy and planning change** | | |
| Targeting high level decision-making | *You'd like to think that there's some changes that happen within local individual health centres, but I think it's got much more potential to be used at that higher level in terms of leverage*. (Academic 3) | Senior manager presented ESP data at inter-agency forum |
| Promoting a strategic approach | *I think the data can be incredibly powerful to shift policy and strategy within the health department, but also in terms of changing the behaviour of practitioners to become adherent with what we see as best practice or evidence-based care.* (Manager 2) | Chronic illness care findings reinforced priorities identified through local CQI processes, informed strategies for brief interventions and referrals for smoking, client self-management |
| Strengthening evidence and opportunities for action | *Now that we know that the priority that we have identified in 'training' - we got that from our consultations with the stakeholders - is actually strengthened with … your report, I can say, "If we truly, really want to improve chronic care, we really need to upskill our health professionals."* (Policy practitioner 1) | Multidisciplinary group used the maternal health findings in submissions for community led risk-reduction programs in infant safety |
| Bringing people together | *I see the role of data as actually bringing strategic people much closer to the frontline practitioners, and then saying to frontline practitioners, "We need to do something about this, help us and we will help you."* (Manager 2) | Findings from ESP and another CQI research project reignited a service's CQI activity, resulting in adjustments in role allocations and service redesign |
| **Theme: Supporting best practice and reflection** | | |
| Supporting CQI activities | *I'm using them. I used them in some feedback recently – you know, "This is a summary of research … we already know these clinical datasets and morbidity. This is the evidence, these are the things that have been measured. This is what we have been looking at and this is what they have found" … for me, it's very useful.* (CQI practitioner 2) | ESP presentations made by people in CQI roles to PHC managers and teams, and at a regional health workshop<br>A facilitated team process to set improvement priorities compared local CQI audit results with aggregated data and priorities identified in ESP reports |

| | | |
|---|---|---|
| Supporting reflection and change | *It made me think outside and my bubble and go, "Wait a minute. This isn't just happening here." And look at how other people are dealing with them, and go, "Can I transport that to here? What can I change to make that work here?" Sometimes you get so snowed under in what you're doing – it's pretty intense where we are. It is good to have those little stops, and go, "No this isn't the only place".* (Clinician 1) | Aggregate CQI data focused PHC team on improving self-management and engagement with clients, better care documentation and client follow up<br>Information in an ESP Maternal Health report prompted cross-checking of the health service's template for recording ante- and post-natal care |
| Affirmation | *It gives us direction on where we should be focusing. … Often, we just carry on with our work and everyday life, and there's very little feedback. And by getting that feedback, we feel a little bit better about what we are doing, and that we are making a difference. It's very important.* (Clinician 4) | Message conveyed by manager to clinicians that their documentation and auditing of patient care made ESP research possible |
| **Theme: Capacity strengthening** | | |
| Building capacity in 1) CQI and 2) population health thinking | 1) *They've provided another layer of information that's stimulated thinking and discussion, that's brought in knowledge and expertise and experience from a broad group. So, it's really enriched the work that we've done.* (CQI role 1)<br>2) *What got me on the road to all this is looking at those gaps and going "Well, why isn't that happening?" I think we have been so focused on what we consider to be best practice, and we're not achieving that across the board. … the bottom line is that we need to get to where the bulk of our people are achieving something.* (Clinician 1) | ESP findings drawn upon when CQI practitioners discussed improvement barriers and strategies with a clinical PHC team<br>Findings on barriers to high quality chronic illness care used to strengthen a proposal to organisational management for community health literacy training<br>ESP report uploaded to reading list for undergraduate nurse training |
| Developing skills in understanding and interpreting data | *I thought, "I don't understand them", and when I looked a bit more, I thought "I'm looking at this graph, I'm going to work it out". And it was quite simple once you took the time about it.* (Clinician 4)<br>*It builds their skills and knowledge, in looking at data and making sense of it, and identifying priorities and strategies out of it. I think it has multi-levelled benefit.* (CQI practitioner 1) | Researcher interrogated findings against her own interpretation of graphed ESP data, to improve understanding and to check whether the overall picture concurred with observations and information recorded at the community level |
| Staff orientation | *For orientation, the report would be part of the core documents that we share with [CQI facilitators].* (CQI practitioner 4) | ESP report given to a new research colleague to enhance understanding of CQI in remote PHC |
| **Theme: Developing new research** | | |
| Developing research based on findings | *I had a vested interest in that report, because it gave me the background knowledge that I would have otherwise had to go looking for. I've referenced those reports on several grants that I've written.* (Academic 2). | Developing CQI research in child health; developing implementation research in maternal health, health promotion |
| Using the research methodology | *The design of the ESP project has absolutely had input to how we've designed our project – as a model. …What we've done is used those reports to logically think through what we need to do, what we need to look at. We've based lots of our information on your graphs … It gave us a good format to start analysing the data.* (Academic 2). | ESP methods informed methods for a collaborative academic/health service project that included audit tool and report development, interviews and stakeholder consultation to prioritise and identify improvement interventions |

| Category | Exemplar quotes | Examples |
|---|---|---|
| **Theme: Multi-level applicability** | | |
| Influencing change at different system levels | *At the micro level it can just start conversations with people individually and gives people permission to talk about [a priority] and to raise it as an issue and on a macro level, it provides this large scale, very hard to argue with, evidence for why action is needed and support from the wider health system, government, funders is needed in terms of resource allocation*. (Academic 3) | An identified ESP priority was social and emotional well-being screening as part of antenatal health checks, countering a general perception that staff were reticent to screen and identifying that resources were needed for staff and community capacity building and for referral services. |
| Supporting a systems approach | *I want to look at a whole systems approach, and that includes this meta-data, not just individual service data but looking at the whole process of care.* (Clinician 7)<br>*You can just see how fixing systems for one area of care, such as childhood anaemia, would work across other areas of care.* (Academic 2) | Reports of ESP results used by CQI practitioners to reinforce the value of CQI as a system improvement method |

ESP: Engaging Stakeholders in Identifying Priority Evidence-Practice Gaps, Barriers and Strategies for Improvement
CQI: continuous quality improvement
PHC: primary health care
Clinician role includes: health centre, service-based and outreach roles
CQI role includes: service-based facilitators, coordination and education roles

**Factors mediating use of ESP project findings: themes and categories with exemplar quotes and examples**

| Category | Exemplar quotes | Examples |
|---|---|---|
| **Theme: commitment to best practice care** | | |
| Valuing data and evidence | *I want best practice. I like to be able to measure that. I'm comfortable with data, and I'm wanting the very best practice - looking at data and wanting to understand where we've been, where we are and where we want to go.* (Clinician 3) | Clinician feedback: Interested in any research that increases understanding of chronic illness care needs and can achieve better outcomes for clients |
| Improving Indigenous health outcomes | *The motivation to take up recommendations and changes, apart from being embedded in policy or, you know, guidelines from above, is also gained by people recognising that it'll make a difference.* (Academic 4) | Comment: Team wants to use ESP reports to inform the best possible primary health care to people living in remote communities |
| **Theme: Perceived relevance** | | |
| To role and work context | *[The report] certainly summarises some of those findings we've identified in relation to CQI and systems failures or lack of systems.* (CQI practitioner 2)<br>*I think that's where system-wide change is everybody and so it's nobody - and that's where it's a bit unclear to me, where that would fit in.* (Clinician 7) | Comment: Findings provide staff on-the-ground, who have limited capacity to access and analyse large-scale data, with important information about PHC for Indigenous populations in other settings |
| Timeliness | *I was feeding back on type 2 diabetes [audit] anyway, so I thought, "Well this is really important, I'll take them out and show them to the practitioners"*. (CQI practitioner 2). | Timing of ESP report coincided with a policy team working on a health department's strategy for that condition |

| | | |
|---|---|---|
| Local versus wider interpretation | *You do need to be a bit careful that a national report hides important jurisdictional differences, and that national decisions are made without reference to more detailed data, which would inform a more locally responsive answer to a system issue.* (Policy practitioner 2). | Comment: ESP reports could be more relevant to services and boards if the graphs representing priority evidence-practice gaps were compared with a service's reports for benchmarking |
| **Theme: Competing pressures** | | |
| Time and workload | *… a lot of (ESP) recommendations are probably not achievable by clinicians on the ground, given time and workload.* (Policy practitioner 2)<br>*The barrier is just the nature of the work. It takes a fair degree of resilience and a work-life balance that you can step out of that and reflect on it and then make the use of such aggregated data forms.* (Manager 2) | Comment: It was challenging for managers to assist staff under pressure to understand why the research was done and what the findings might mean for daily work |
| Staff shortages and turnover | *Retention of staff is another really big issue. It has been for a long time. It's very hard to have a PHC mindset when you're there for 3 months, 6 months, 12 months, and that's all you can manage to keep people. How do you talk 10-year goals?* (Clinician 8) | Manager raised the multiple challenges experienced by newly appointed staff working in complex cross-cultural settings and teams. They need to learn new systems and form productive relationships with colleagues who come and go in remote settings |
| **Theme: Organisational environment for change** | | |
| Role of managers, organisational change | *The big thing is, for traction you've got to go through the managers, to set the agenda, and then everybody else comes on board. I think at the moment it's not connected - this group's doing it, that group's not. And I don't think anybody's fault it's just the restructuring and everything that's been going on.* (Clinician 1) | Comment: While managers agree with identified priorities and are supportive of stronger engagement with CQI, it can be difficult in stressed healthcare systems to make it happen |
| PHC approach v's acute care focus | *Most clinics are very acute driven mini emergency departments and PHC comes second.* (Clinician 8) | Comment: Findings reinforce the need and urgency of focusing services on preventing chronic disease in younger generations |
| **Theme: Presentation and useability** | | |
| Report formats for different audiences | *It's getting the information out there in easily digestible form, so that people have an option to look at a summary that says these are the key areas or can drill down and look in more depth - people in different roles want different levels of information.* (CQI practitioner 1) | Feedback: The background and detail in the full report enabled a more comprehensive understanding of the project by a researcher |
| Accessible information | *Just by seeing this [plain language summary], you can kind of go "These are areas we need to target", and that's great. … when you have a group, you can go along and say, "It's recognised here that it's an issue … so, we need to continue with it. What can we do?"* (Clinician 6) | Feedback: The researchers writing the reports needed to think in the language that clinicians and managers think in |
| Support for learning | *When people see the data, and particularly if someone talks them through the data and they start to understand what that means, and they're able to then look at it themselves - they're able to then go and talk to other people. That's the process of really starting to drive change.* (Manager 1) | CQI training participants interrogated the graphs, discussed the number of services represented, what it meant across the country, where their work was making a difference, and what needed to improve at a wider level |
| **Theme: Credibility of research findings** | | |

| | | |
|---|---|---|
| Research history and methodology | *I think the credibility of this process is very strong and the fact that it supports change - people hearing about the pedigree of this project, because it did begin a long time ago and this final outcome, of making recommendations for change, is not something that's come about easily. So, the story, the narrative, is one that's of very strong credibility* (Academic 4) | Feedback: The consultative process of gathering and presenting interpretive input was unusual, compared with the way most data are analysed and reported to research users |
| Data currency, benefits and limitations | *Oh, I'm using it already. … And I imagine that other researchers are as well, because it's up-to-date information on what's happening at the moment. …It's very current, relevant data, so it's like getting it live. That's a real big positive.* (Academic 2)<br>*If you target the upper management it's fine, but if you're going to target it to become a more working document for CQI, then. … we need the next level between the upper management and the service, which is middle management.* (CQI practitioner 5) | Comment: ESP data are hard evidence gathered over years of the CQI research and support program. They reflect the reality of clients and staff and can help resource local CQI processes |
| **Theme: Facilitation and communication** | | |
| Interactive process | *I do like [the reports] presenting the stakeholder priorities back to the stakeholders when asking about barriers and enablers. "This is what you've said, we've taken that on board. This is the next step. What can we do about it?" I think that's really powerful to acknowledge the consultation and to reassure people that their voices have been heard.* (Academic 3) | Comments: It was important to have access to someone who could help make sense of the data. The ESP provided another layer of information that stimulated thinking and discussion and brought in knowledge and expertise from a wider group |
| Methods and paths of communication | *I think it would be really worthwhile to be thinking about how we can get this information out there and continue the discussion…. having people committing the time to doing it, we want to make sure the information is accessible in lots of different ways.* (CQI practitioner 1) | Request for a one-page plain language summary to make the findings more intelligible, useful, and help facilitate CQI processes with teams. Comment that summary a useful resource for discussing health promotion with community groups |

CQI: continuous quality improvement
ESP: Engaging Stakeholders in Identifying Priority Evidence-Practice Gaps, Barriers and Strategies for Improvement
PHC: primary health care
Clinician role includes: health centre, service-based and outreach roles
CQI role includes: service-based facilitators, coordination and education roles
